# One-Drop Self-Assembly of Ultra-Fine Second-Order Organic Nonlinear Optical Crystal Nanowires

**DOI:** 10.1186/s11671-019-3103-y

**Published:** 2019-08-07

**Authors:** Tian Tian, Bin Cai, Qingqing Cheng, Cheng Fan, Yanyan Wang, Gongjie Xu, Fuxing Gu, Feng Liao, Okihiro Sugihara, Eiji Hase, Takeshi Yasui

**Affiliations:** 1 Shanghai Key Lab of Modern Optical System, Ministry of Education, University of Shanghai for Science and Technology, Shanghai, 200093 China; 2 Graduate School of Engineering, Utsunomiya University, Utsunomiya, 321-8585 Japan; 3 Department of Mechanical Engineering, University of Tokushima, Tokushima, Japan

**Keywords:** Nanowires, DAST, Nonlinear, Self-assembly

## Abstract

In this study, we propose a one-drop self-assembly method, which proved capable of successfully preparing 4-N, N-dimethylamino-4′-N′-methyl-stilbazolium tosylate (DAST) single-crystalline nanowires (NWs). The apparent roughness of the DAST NWs was determined to be less than 100 pm by using a high-resolution atomic force microscope, indicating their ultrafine quality. The DAST NWs also exhibited excellent nonlinear optical properties, including two-photon excited fluorescence and second harmonic generation, which could enable the production of low-cost, low-power-consumption wideband wavelength conversion devices. Thus, the described method may provide a new avenue for organic NW fabrication.

**Electronic supplementary material:**

The online version of this article (10.1186/s11671-019-3103-y) contains supplementary material, which is available to authorized users.

## Introduction

Second-order organic nonlinear optical (NLO) materials have ultra-fast electro-optic response times as well as very large bandwidths and NLO coefficients, and therefore, have been intensively researched for a wide range of applications related to electrical/optical signal transduction, optical switching, phased-array radar, analogue/digital conversion, terahertz signal generation, and digital signal processing [1, 2]. Organic ionic 4-N, N-dimethylamino-4′-N′-methyl-stilbazolium tosylate (DAST) crystals are recognized as benchmark organic NLO crystals due to their electro-optic coefficients *γ*_11_ = 55 ± 80pm/V at 1315 nm , high-NLO susceptibilities *χ*^(2)^(−2*ω*, *ω*, *ω*) = 580 ± 30pm/V at 1535 nm, and low-dielectric constants (5.2, 10^3^–10^5^ kHz) [3, 4] and consequently are researched intensively [5–9]. However, the applications of DAST crystals are limited due to their deficient quality and the difficulty of fabricating DAST crystal optical waveguides using the traditional “top-down” approach. Meanwhile, self-assembly, a bottom-up technique, is becoming a powerful method for fabricating micro/nanoscale one-dimensional (1D) structures and is promising for the production of miniaturized integrated electronic, optoelectronic, and photonic devices [10–12]. For organic materials, the self-assembly driving forces can originate from interactions such as coordination bonding, aromatic π−π stacking, hydrogen bonding, Van der Waals forces, and electrostatic interactions [13–15]. Although many organic materials have been successfully employed to synthesize second harmonic generation (SHG) active 1D crystalline nanostructures, their second-order susceptibilities are still far lower than those of organic NLO crystals with large dipole moments [16]. In this study, we develop an environmentally friendly one-drop self-assembling method for DAST NWs fabrication. We separate crystal seeds preparation and growth process by substrate-supported rapid evaporation crystallization (SSREC) [17, 18] and saturated vapor cultivation respectively. In this way, we can easily obtain ultra-fine single-crystalline DAST NWs with good NLO properties.

## Methods

The DAST powder (Daiichi Pure Chem. Co. Ltd.), methanol (99.9%, Surper Dry, with molecular sieves, water ≦ 30 ppm, J&K Seal), and the surfactant (cetyltrimethylammonium bromide, CTAB, TCI) were used directly without further purification.

### Preparation

Firstly, 30 mg of DAST powder and 10 mg of the surfactant were dissolved in 5 mL of methanol. Next, 100 μL of this DAST-CTAB methanol solution was diluted with 10 mL of methanol (DAST concentration of approximately 0.146 mM, ) and stirred for 0.5 h to obtain a homogeneous solution (for more details, see Additional file 1: Figure S1).

### Characterization

The DAST NW morphology was studied using an optical microscope (Imager.A2m, Zeiss), BTEM (Tecnai G2 SpiritBiotwin), SEM (Nanolab600i, Helios, Quanta 200, Fei), and AFM (MultiMode8, Bruker). The UV-Vis spectra were obtained using a fiber spectrometer (Nova, Idea Optics). The crystal structure of the NW was examined using XRD (D/Max 2550 V, Rigaku). The two-photon excited fluorescence (TPEF) was excited using a 1064 nm cw laser (MIL-III-1064-1W, CNI), the images were obtained using an optical microscope (DS-RI2, Nikon), and the emission spectrum was measured using the fiber spectrometer.

The second harmonic generation (SHG) polarization dependence of the DAST NCs was measured using a homemade SHG microscope. A 1250-nm femtosecond laser (Insight DeepSee, Spectra-Physics) with a wavelength of 1250 nm, the repetition rate of 80 MHz, and a pulse width of 130 fs were employed as the light source (for more details, see Additional file 1: Figure S1).

## Results and Discussion

**Fig. 1:**
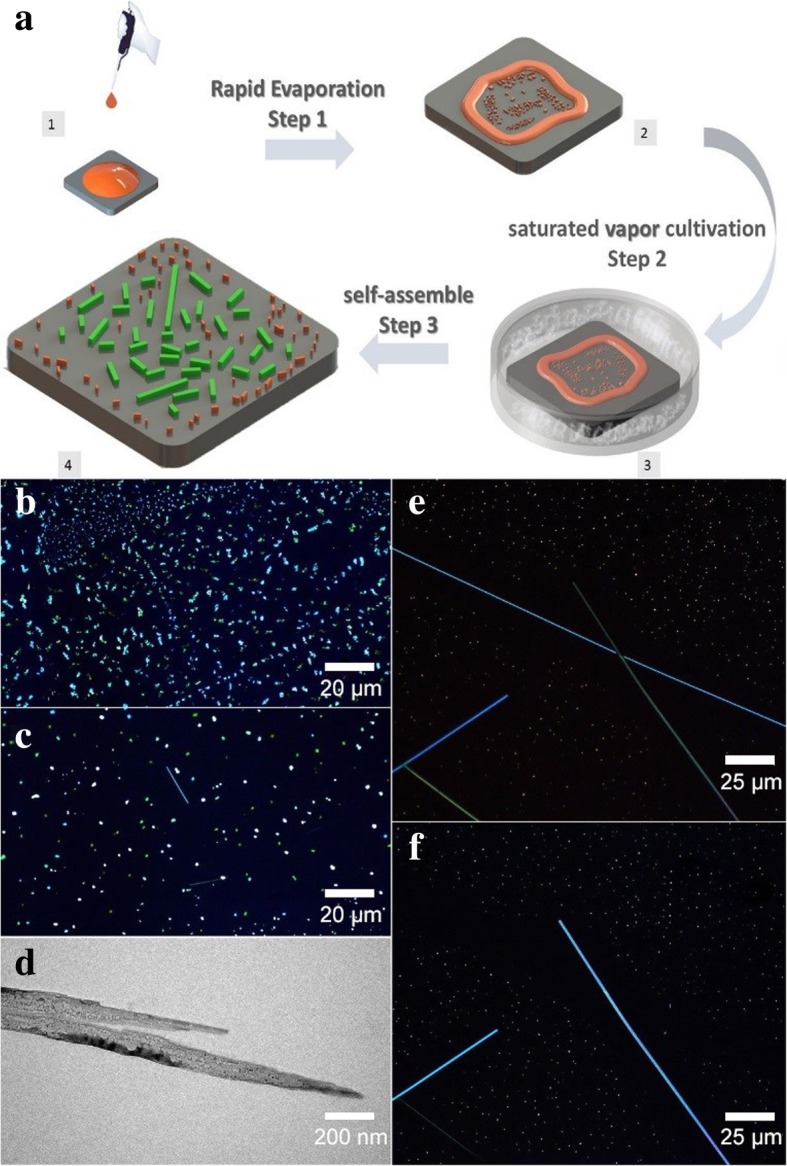
**a** The schematic of the one-drop self-assembly method. **b** The fluorescence image of DAST crystal after the SSREC process. **c** After 40 min of cultivation in a methanol atmosphere. **d** The BTEM image of unfinished DAST NW. **e** After 2.5 h of cultivation in a methanol atmosphere. **f** After 2.5 h of cultivation in a methanol atmosphere with the cross-polarizer rotated by 90°

A schematic of the one-drop self-assembly method is shown in Fig. 1a. First, the hydrophilic substrate was placed on a hot plate and heated to 80 °C. Then, 100 μL of the 0.146 mM DAST-CTAB methanol solution was dropped onto the heated hydrophilic substrate and heated continuously for 20 s. As the methanol solvent spread and evaporated, the DAST nano/microcrystals (NC/MCs, orange color) were rapidly deposited on the substrate shown in step 1, Fig. 1a. In step 2, the substrate was placed into a culture dish and sealed with approximately 0.1 mL of methanol solvent for the wet-cultivation process. After ~ 3 h of cultivation at room temperature, the DAST NWs (green color) were obtained in step 3. The morphological evolution of the DAST crystals is shown in Fig. 1b–f. The DAST crystals as-deposited appear as micro-flakelets with a relatively high density, and no wires can be observable as shown in Fig. 1b. And then after 40 min of cultivation in the atmosphere of saturated methanol vapor pressure, at room temperature, the shorter DAST rods begin to appear, shown in Fig. 1c.The slice-shaped crystals are smaller than the crystals in Fig. 1b. Furthermore, after 2.5 h of cultivation, some longer DAST crystal wires show up, see Fig. 1e. The DAST wires have uniform widths generally with several hundred micrometers long; some of them could even be longer than 1 mm. There is a microscope image of the DAST wires with the polarizer rotated by 90° in Fig. 1f. The entire tilted wires appear to have been changed from their maximized anisotropy birefringence (brightest) states to their minimal (extinguished) states. This alteration implies that the single crystalline structures of the fabricated DAST wires are highly uniform. Meanwhile, large DAST crystal particles no longer appear, and instead, small crystal dots are visible. Furthermore, the crystal dot density near the DAST wires is obviously lower than the density further away.

Thus, we expect that the DAST wire formation process was as follows. After the SSREC process, small DAST crystals were deposited on the substrate. When they were put into the methanol atmosphere, the DAST crystals absorbed the methanol and partially dissolved into it, resulting in NC/MCs surrounded by a DAST-saturated methanol solution. The self-assembling driven force could originate from the huge dipole moments of NC/MCs [19–21]. For instance, an MC with 0.35-micron diameter may have a dipole moment magnitude as large as ~ 4.5 x 10^4^ D. Meanwhile, the methanol solution acts as a lubricant and can facilitate the motion of the DAST NC/MCs. Due to the electrostatic interaction and the lubrication provided by the methanol solution, the DAST NC/MCs undergo self-assembly, yielding DAST NWs. As an evidence, a biology transmission electron microscope (BTEM) image of an unfinished DAST NW is shown in Fig. 1d, plenty of NCs gathered in the NW can be confirmed. As the DAST NWs grow, they continually absorb the nearby DAST solution via the capillary effect. Consequently, as is evident from Fig. 1d, e, the residual crystal dot density near the wires is lower than that further away.

**Fig. 2:**
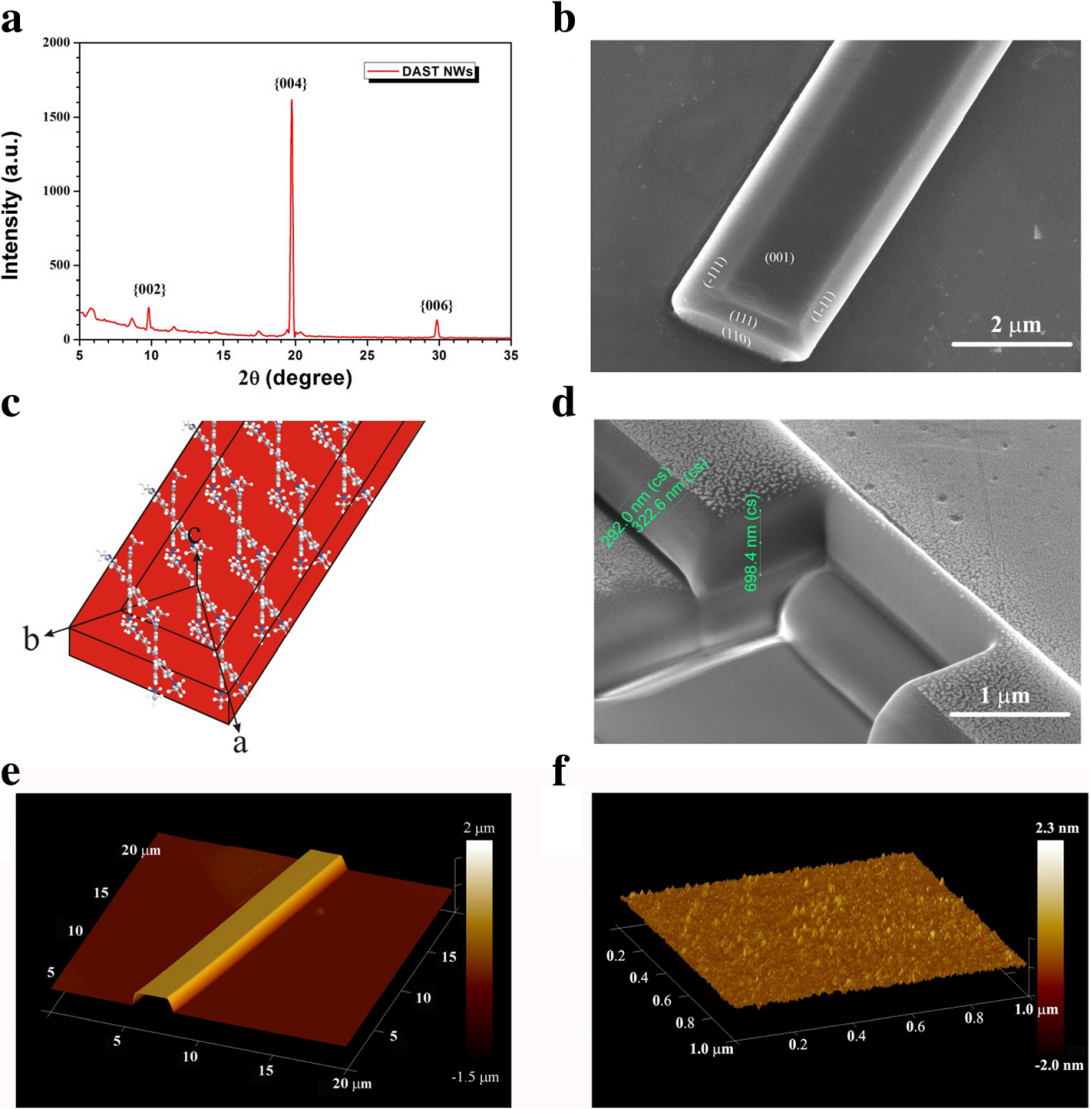
**a** The XRD pattern of DAST NWs. **b** SEM image of a DAST NW from the [001] perspective, where, based on the typical characteristics of bulk crystal growth, the [-111], [111], [1-11], and [110] faces can be identified. **c** DAST molecular alignment in the NW, where the tosylate anions have been omitted for clarity. **d** The cross-section of a DAST NW cut by a focused ion beam. **e** AFM image of a DAST NW. **e** The surface roughness of a DAST NW, as determined using an AFM

An X-ray diffraction (XRD) pattern of DAST NWs is presented in Fig. 2a. By referring to the crystal cell parameters of bulk DAST crystals (monoclinic Cc space group, point group *m*, *a* = 10.365 Å, *b* = 11.322 Å, *α* = *β* = 90°, and *γ* = 92.24°) [4], the diffractive peaks near 10°, 20°, and 30° corresponding to the [002], [004], and [006] faces of the DAST crystals, respectively. It means that DAST NWs grown on the substrate have the [001] orientation with the *a*- and *b*-axes along the film plane. The morphologies of the DAST NWs were further studied using an SEM. An SEM image from the [001] perspective is shown in Fig. 2b. Based on the characteristics of bulk DAST crystal growth, the [111], [-111], [1–11], and [110] end faces can be identified easily (see Fig. 2). The single-crystalline DAST NW has a belt-like morphology, with a typical width of 1.5 ± 0.5 μm and thickness of 0.8 ± 0.4 μm. According to the NW’s crystal orientation, the alignment of the DAST molecules in the NW can be depicted as in Fig. 2c, the tosylate anions have been removed for clarity. For the DAST, bulky crystal directly grows from saturated DAST solution, the growth speed along the crystallographic *a*-axis is the fastest, in other words, in the [100] direction [22]; however, in the case of self-assembly, the [110] direction has priority. Thus, there is a different mechanism drive the NW fabrication. We expect that the NW formation begins with DAST NC/MC self-assembly due to the electrostatic force. For a four-molecule model, the dipole moments along the *a*-, *b*-, and *c*-axes in a DAST crystal lattice are 158.2 D, 141.2 D, and 121.0 D, respectively [18]. The growth along the *c*-axis was limited due to the configuration of the present experiment, so the NWs could only grow along the direction of the vector sum of the dipole moments of the *a*- and *b*-axes. Since the dipole moments along the *a*- and *b*-axes are similar, the [110] direction become preferred NW growth direction.

To investigate the internal quality of the DAST NWs, we used an electron beam to cut an NW, whose cross-section was shown in Fig. 2d. The white dots on the NW are silver nanoparticles that were deposited to increase the conductivity of the NW. The roughness of the cut cross-section is similar to as-grown DAST crystals, and no defect can be detected at this scale. We further investigated the DAST NW morphology by using a high-resolution atomic force microscope (AFM) to obtain the surface information directly without disturbing the metallic silver nanoparticles. As shown in Fig. 2e, the NW has a clear belt configuration with the [001] face as the flat top surface, which is consistent with the SEM results. By zooming in on the top surface of the DAST NW using the AFM, we obtained the morphology of a 1000 x 1000 nm^2^ region of the [001] face, which is presented in Fig. 2f. According to the results, an average roughness of DAST NW is about ~ 85 pm, which is even smaller than that of a graphene monolayer on a SiO_2_ substrate [23]; thus, an ultra-flat DAST NW crystal was realized. For a low-loss optical waveguide, its surface roughness must be less than 10 nm. Obviously, our DAST NWs were of much higher quality than required.

**Fig. 3:**
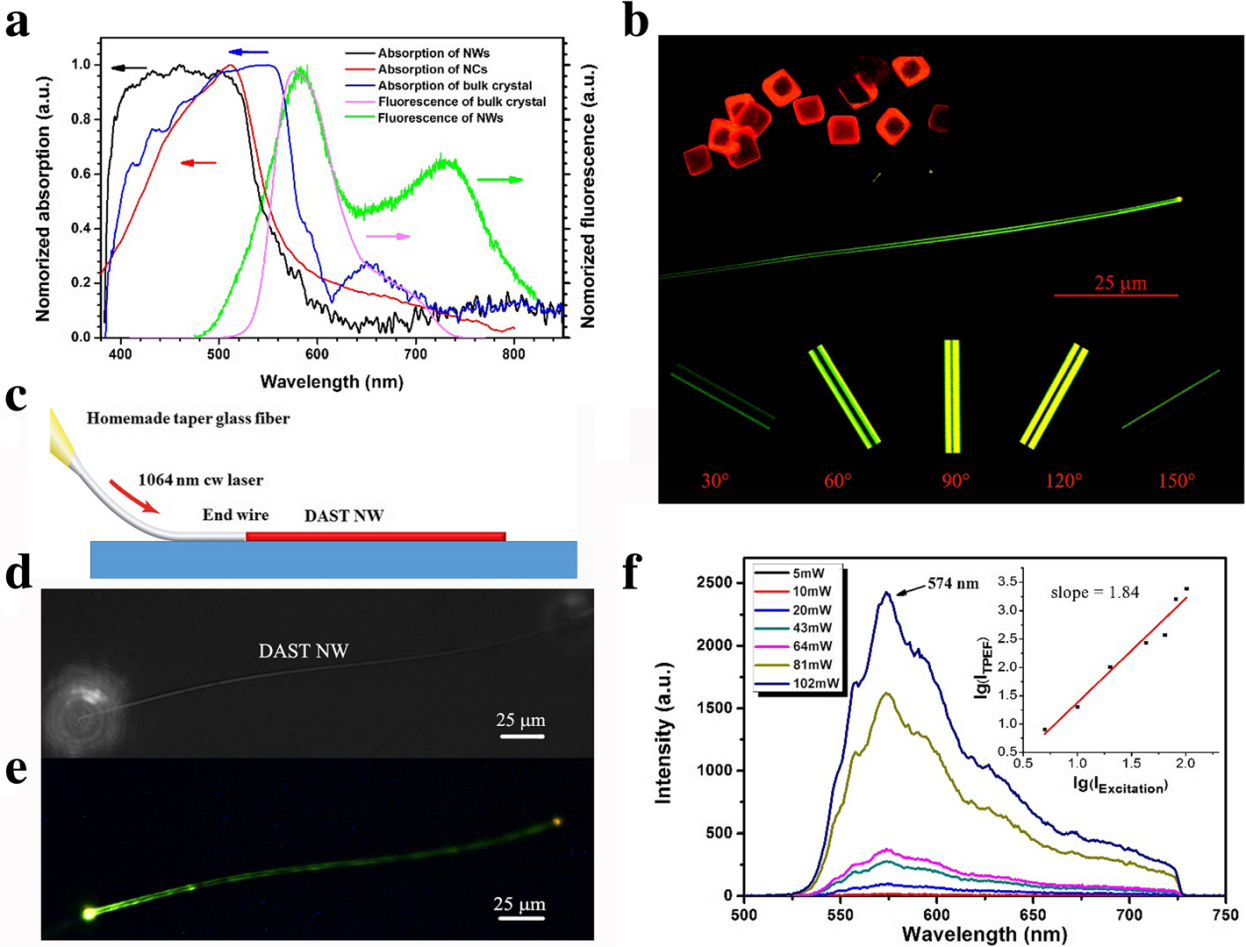
**a** Absorption spectra of DAST NWs, NCs, and bulk crystals and fluorescence spectra of DAST NWs and bulk crystals excited by a 407 nm laser. **b** Fluorescence images of DAST MCs (top left), a DAST NW (center), and the DAST NW at different polarization angles (bottom). **c** Optical setup for TPEF measurements. **d** Top view of a DAST NW with 1064 nm laser input. **e** TPEF image of a DAST NW upon irradiation by the 1064 nm cw laser. **f** TPEF spectra of the DAST NW at different input laser intensities, where the inset shows the logarithm of the TPEF intensity as a function of the logarithm of the input laser intensity

The UV-Vis absorption spectrum of the DAST NWs is compared with those of the bulk crystal, NC, and solution states in Fig. 3a. Due to the unique 1D conformation of the NW, its absorption spectrum (black line) is obviously different from those of the other states. In methanol solution, an absorption peak at ~ 476 nm [8, 17] is originated from the π-conjugation system of DAST cation. Upon crystallization, due to the electronic transition of the π-π conjugated system of the cation, the absorption band will expand toward hypsochromic and bathochromic shifts (i.e., blue and red shifts, respectively). The bathochromic shift originates from the J-aggregation of the chromophore cations along the *a*-axis of the crystal in a head-to-tail stacking mode, while the hypsochromic shift originates from the H-aggregation in a face-to-face stacking mode [24, 25]. The NCs exhibit only a slight expansion in the blue and red directions, as indicated by the red line in Fig. 3a with an absorption peak at ~ 512 nm, which reflects the size limitations of the two aggregation directions. In the bulk crystal case, the J- and H-aggregation lengths are significantly expanded, so the bulk crystal spectrum has the broadest absorption band, which extends from 350 nm to 750 nm, with an absorption peak at ~ 550 nm, as shown by the blue line in Fig. 3a.

In contrast, the DAST NWs, due to their well-structured 1D conformation, have an absorption band from ~ 380 nm to 600 nm, as indicated by the black line in Fig. 3a. They exhibit only a slight extension in the red direction and more significant extension in the blue direction. In other words, the NW growth does not proceed along the *a*-axis (J-aggregation direction), which is consistent with the NW morphology results. Owing to the 1D structure of the NWs, the H-aggregation is greatly enhanced, causing the NWs to exhibit similar absorption on the blue side. The fluorescence spectrum of the DAST NWs was also different from bulky DAST crystals. The spectra of DAST bulk crystals and NWs are depicted in Fig. 3a in pink and green, respectively. Since shortening the J-aggregation lengths of chromophores will cause the blue-shifted of the fluorescence spectrum [26], the DAST NWs have a spectrum more hypsochromic than that of the bulk crystals. In contrast, the cut-off wavelength on the short-wavelength side of the DAST NW spectrum is blue-shifted by ~ 30 nm. There is a new peak at ~ 730 nm, which may originate from the Fabre-Perot resonance [27] of the DAST NWs’ linear structures. The fluorescence microscopy images in Fig. 3b confirm these differences.

DAST crystals with size on the order of micrometers are depicted in the upper left and appear orange upon irradiation by blue light. However, the DAST NW emits yellow-green light when irradiating under the same conditions. Besides, the end of the NW is distinctly brighter than its body, which implies that the NW confines light well; in other words, the fluorescent light was confined and enhanced by this waveguide structure [27]. The fluorescence also exhibits very strong polarity, as illustrated at the bottom of Fig. 3b, which shows polarized microscope images of a single NW rotated at various angles. Clearly, the fluorescence is altered as the rotation angle changed, which illustrates that DAST NW has strong polarized property.

We launched a 1064-nm continuous wave (cw) laser into a DAST NW through a home-made taper fiber to observe its propagation characteristics. The pump light was launched using a butt-coupling technique [26] with the setup illustrated in Fig. 3c. A top view of the propagation is shown in Fig. 3d. Since the DAST NW has ultra-fine crystal quality, there is no distinct scattering from its side walls. We could not evaluate the propagation loss because it is difficult for the NW’s cut-back to change the propagation distances. The NW exhibited fluorescence even when it was irradiated by the 1064 nm cw laser. A fluorescent image of the DAST NW is presented in Fig. 3e, which emits yellow-green light which is very similar to when irradiated with blue light. We can easily confirm the 1064 nm laser propagation path since the NW absorbs the laser light and emits fluorescence. The 1064 nm laser light and the fluorescent light are well confined in the NW structure, as evidenced by the fact that the end of the NW is brighter than its side walls. The fluorescence intensity increases as the laser input increases, as shown in Fig. 3f. The dependence of the two-photon excited fluorescence (TPEF) intensity (*I*_TPEF_) on the excitation intensity (*I*_Excitation_) was analyzed by taking the logarithm (lg) of each quantity. The plot of lg *I*_Excitation_ vs. lg *I*_TPEF_ appears in the inset of Fig. 3f, where the slope *k* of the fitting line is 1.84, near 2, it demonstrates the quadratic of the TPEF dependence on the excitation intensity in this measurement range. Notably, the TPEF signals were collected from the top of the NW, which was perpendicular to the light propagation direction. Along the propagation direction, the spectrum could be significantly different due to the resonance in the waveguide. The SHG signal was not observable using this setup for the following reasons: the exciting laser was launched from the end of the NW; the SHG signal collected from the top of a well-confined waveguide structure [26] is weak; the phase is mismatched in an as-grown DAST NW; the strong TPEF masked the SHG signal; and the SHG signal was in the crystal absorption band.

**Fig. 4:**
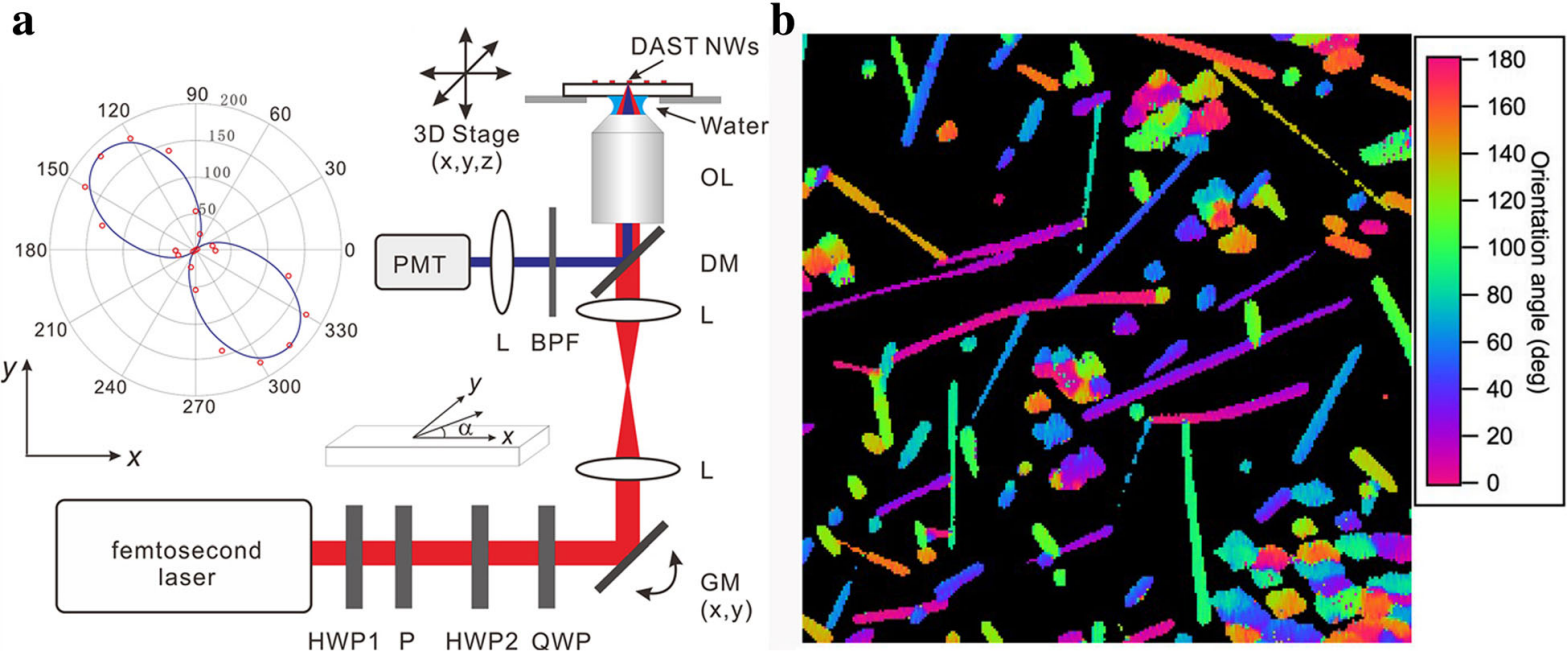
**a** The SHG microscopy setup, where L is a lens, OL is an objective lens, DM is a dichroic mirror, GM is a galvanometer mirror, P is a polarizer, HWP is a half-wavelength polarizer, QWP is a quarter-wavelength polarizer, BPF is a band-pass filter, and PMT is a photomultiplier tube. The inset is a typical plot of the SHG intensity of a NW as a function of the incident laser polarization angle, where the red dots indicate the experimental data, and the solid blue lines represent theoretical fits. **b** SHG image of DAST NWs and their orientation angles, the field size is 170 x 170 μm

The crystalline features of the DAST NW were further investigated by performing SHG microscopy; the setup is shown in Fig. 4a. The SHG responses as functions of the incident laser polarization angle were collected. The inset of Fig. 4a presents a typical polar plot, where the red dots indicate the experimental data. The intensities of the parallel and vertical SHG components, *I*_*x*_ and *I*_y_, respectively, can be written as

$$ {I}_x^{2\omega }=A\cos 4\alpha +B\cos 2\alpha +C $$ (1)

and

$$ {I}_y^{2\omega }=\frac{K}{2}\left(-\cos 4\alpha +1\right) $$ (2)

Where *α* is the angle between the laser polarization and the long axis of the NW; *A*, *B*, and *C* are parameters related to the material; and *K* is a constant merging various parameter [28]. The solid lines in the inset of Fig. 4a represents the theoretical fits obtained using Eqs. (1) and (2). The observed SHG response has a two-lobe pattern, indicating that the anisotropy of the SHG is attributable to the intrinsic orientation of the DAST crystal. Since the SHG signal is emitted laterally from the NW [29], it is difficult to evaluate the second-order susceptibility tensors in the NW for this setup.

A DAST NW SHG image with a field size of 170x170 μm is shown in Fig. 4b where the colors represent the NW orientation angles of the crystals. The DAST NWs and MCs are distributed throughout the depicted area. Both the NWs and the crystals emit SHG signals, which indicate that both have SHG active crystal structures and similar NLO properties. The SHG signals from the NWs are relatively uniform, which indicates the high quality of the NWs.

## Conclusions

In this study, we demonstrated a one-drop self-assembling method for the DAST NWs preparation. The DAST NWs have SHG active crystal structure with very strong TPEF. The DAST NWs were observed to be single-crystalline, to have few defects, and to be well-faceted with ultra-fine surface roughness of 85 pm, which is highly beneficial for integrated device fabrication. Moreover, the method is highly efficient, the material requirement can be lower to microgram level (only 6 μg is needed in our fabrication process); thus, it is very eco-friendly.

## Additional Files

Additional file 1: **Figure S1.** DAST NWs preparation. (DOC 2375 kb)

## Data Availability

All the data are fully available without restrictions.

## Abbreviations

AFM: Atomic force microscope
BTEM: Biology transmission electron microscope
CTAB: Cetyl trimethyl ammonium bromide
DAST: 4-N, N-dimethylamino-4′-N′-methyl-stilbazolium tosylate
MCs: Microcrystals
NCs: Nanocrystals
NLO: Nonlinear optical
NWs: Nanowires
SEM: Scan electron microscope
SHG: Second harmonic generation
SSREC: Substrate-supported rapid evaporation crystallization
TPEF: Two-photon excited fluorescence
XRD: X-ray diffraction
