# Supplementary material for: One-Drop Self-Assembly of Ultra-Fine Second-Order Organic Nonlinear Optical Crystal Nanowires
Source: Nanoscale Res Lett. 2019 Aug 7;14:269. doi: 10.1186/s11671-019-3103-y (PMC6686028; doi:10.1186/s11671-019-3103-y)
Supplement: Supplementary file 1 — Figure S1. DAST NWs preparation. (DOC 2375 kb) [file 11671_2019_3103_MOESM1_ESM.doc]

**Supportive information**

**One-drop Self-Assembly of Ultra-Fine Second-Order Organic Nonlinear Optical Crystal Nanowires**

**Method Section**

**Materials**

The DAST powder (Daiichi Pure Chem. Co. Ltd.), methanol( 99.9%, SurperDry, with molecular sieves, water≦30 ppm,J&Kseal.) and the surfactant CTAB, (TCI) were used directly without further purification.

**DAST NW preparation.** Firstly, 30 *m*g of DAST powder and 10 *m*g of the surfactant (cetyl trimethylammonium bromide, CTAB, TCI) were dissolved in 5 *m*L of methanol. Next, 100 *μ*L of this DAST–CTAB methanol solution was diluted with 10 *m*L of methanol (DAST concentration of approximately 0.146 *m*M) and stirred for 0.5 h to obtain a homogeneous solution. The hydrophilic substrate was placed on a hot plate and heated to 80 °C. Then, 100 *μ*L of the 0.146 *m*M DAST–CTAB methanol solution was dropped onto the heated hydrophilic substrate and heated continuously for 20 s. We can see the DAST NC/MCs (the orange color) aggregated together as shown in step 1 of Figure.1, and then they were put into the methanol atmosphere as shown in step 2 of Fig. 1. As the methanol solvent spread and evaporated, the DAST NC/MCs were rapidly deposited on the substrate. Finally, the substrate was placed into a culture dish and sealed with approximately 0.1 *m*L of methanol solvent for the wet-cultivation process. In the methanol atmosphere, the DAST particles absorbed the methanol and partially dissolved into it, resulting in NC/MCs self-assembled into DAST NWs (the green color) as shown in step 3 of Fig.1.


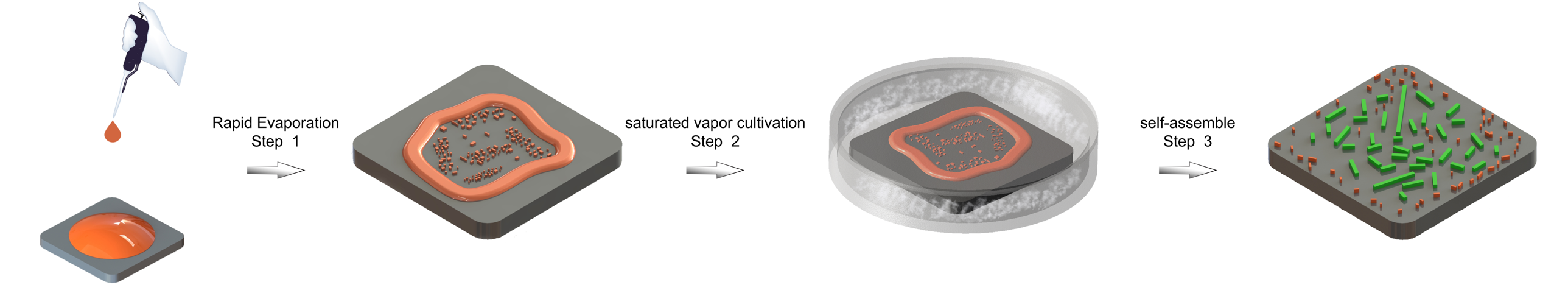


**Fig. 1** Schematic of the process for fabricating DAST NWs.

**Characterization of DAST NW.** The DAST NW morphology was studied using an optical microscope (Imager.A2m, Zeiss), SEM (Nanolab600i, Helios and Quanta 200, Fei), BTEM (Tecnai G2 SpiritBiotwin) and AFM (MultiMode 8, Bruker). The UV-Vis spectra were obtained using a fibre spectrometer (Nova, Idea Optics). The structure of the NW was examined using XRD (D/Max 2550V, Rigaku). The TPEF was excited using a 1064 nm CW laser (MIL-III-1064-1W, CNI), the images were acquired using an optical microscope (DS-RI2, Nikon), and the emission spectrum was measured using the fibre spectrometer. There are not preparation for AFM and AFM, we just put the substrate on the sample table (The substrate size is 1 cm × 1 cm).

**Preparation of SEM sample.** Due to the DAST crystal is a semiconductor with poor electrical conductivity, spraying silver can enhance its conductivity. So we applied magnetron sputtering equipment(microhezao GVC-2000) for SEM sample preparation. The specific parameters are as follows: spraying silver for 15s, the current is 12 MW and magnetron vacuum is 3 Pa. The thickness of silver particles on the surface of the sample was about 50nm.

**SHG imaging of DAST NW.** The polarization dependence of the DAST NCs was measured using a homemade SHG microscope (Figure 4(a)). A 1250 nm femtosecond laser (Insight DeepSee, Spectra-Physics) with a wavelength of 1250 nm, the repetition rate of 80 MHz, and a pulse width of 130 fs was employed as the light source. HWP1 and P were utilized to control the output intensity of the laser, while HWP2 and QWP were used to tune the polarization direction of the excitation laser source. The objective lens had a numerical aperture of 0.90 and a 2 mm working distance. A dichroic mirror and BPF (612–638 nm) were employed to remove the excitation laser light and TPEF signal. The SHG signal was detected using a photomultiplier tube (PMT, H7421-40, Hamamatsu). The polarization dependence of the incident laser was investigated by rotating the polarization angle 15° at each incident angle of the excitation laser.


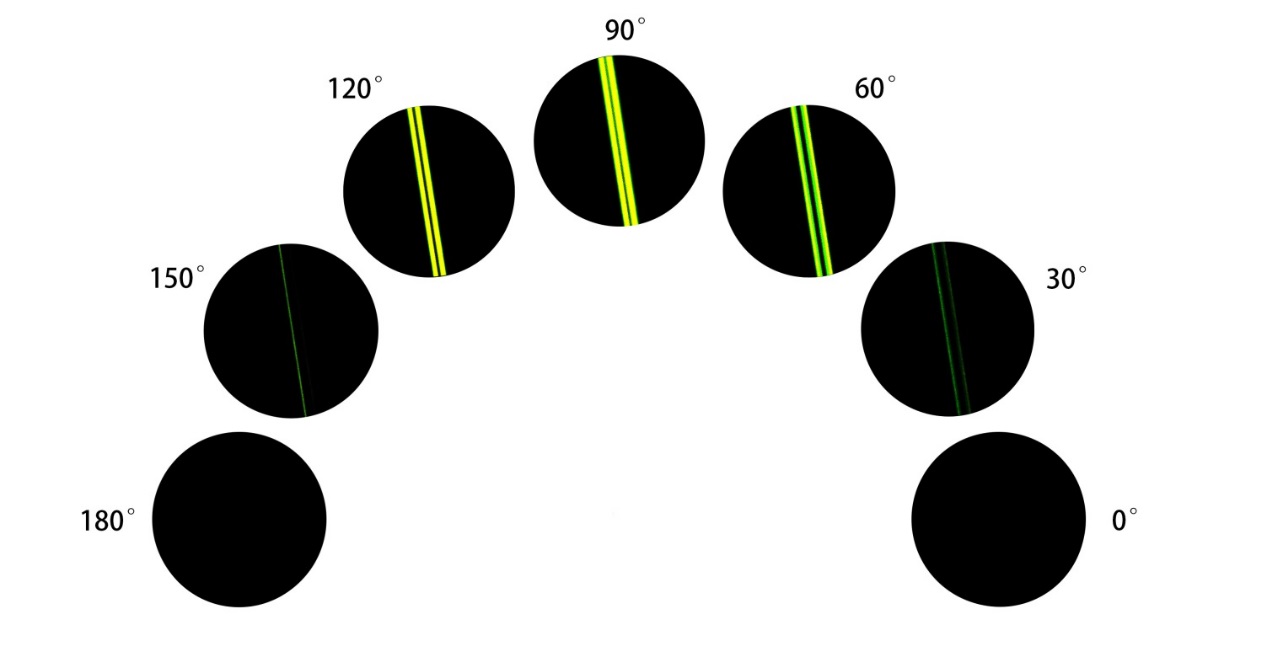


**Fig.2** The fluorescence images with the difference-angles polarization direction
